# Pressure Pain Hypersensitivity and Ultrasound Changes in the Radial Nerve in Patients with Unilateral Lateral Epicondylalgia: A Case–Control Study

**DOI:** 10.3390/diagnostics13152488

**Published:** 2023-07-26

**Authors:** Ignacio Cancela-Cilleruelo, Jorge Rodríguez-Jiménez, César Fernández-de-las-Peñas, Joshua A. Cleland, José L. Arias-Buría

**Affiliations:** 1 Escuela Internacional de Doctorado, Universidad Rey Juan Carlos, 28922 Alcorcón, Spain; ignacio.cancela@urjc.es; 2 Department of Physical Therapy, Occupational Therapy, Rehabilitation and Physical Medicine, Universidad Rey Juan Carlos, 28922 Alcorcón, Spain; jorge.rodriguez@urjc.es (J.R.-J.); joseluis.arias@urjc.es (J.L.A.-B.); 3 Doctor of Physical Therapy Program, Department of Public Health and Community Medicine, Tufts University School of Medicine, Boston, MA 02111, USA; joshua.cleland@tufts.edu

**Keywords:** lateral epicondylalgia, pressure pain threshold, cross-sectional area, radial nerve

## Abstract

Some authors have proposed the potential role of the radial nerve in lateral epicondylalgia. The aims of this study were to investigate the presence of pressure pain hyperalgesia and nerve swelling (increased cross-sectional area) assessed with ultrasound imaging on the radial nerve in people with lateral epicondylalgia, and to investigate if an association exists between pressure pain sensitivity and cross-sectional area. A total of 37 patients with lateral epicondylalgia (43% women, age: 45.5 ± 9.5 years) and 37 age- and sex-matched pain-free controls were recruited for participation. Pressure pain thresholds (PPTs) were assessed bilaterally on the radial nerve at the spiral groove, the arcade of Frohse, and the anatomic snuffbox in a blinded design. Further, the cross-sectional area of the radial nerve at the spiral groove and antecubital fossa was also assessed. The results demonstrated lower PPTs on the radial nerve of the affected side in individuals with lateral epicondylalgia as compared with the unaffected side (*p* < 0.01) and with both sides in healthy controls (*p* < 0.001). Additionally, the cross-sectional area of the radial nerve on the affected side in patients was higher compared with the unaffected side (*p* < 0.01) and both sides in healthy controls (*p* < 0.001). The cross-sectional area of the radial nerve at the spiral groove was negatively associated with PPTs over the radial nerve at the spiral groove (r = −0.496, *p* = 0.002) and positively associated with function (r = 0.325, *p* = 0.045). Our findings revealed generalized pressure pain hyperalgesia and also nerve swelling of the radial nerve in people with lateral epicondylalgia, suggesting the presence of a widespread sensitization of nerve tissues in this population. The radial nerve could represent a potential peripheral drive to initial and maintain altered pain processing in lateral epicondylalgia.

## 1. Introduction

Lateral epicondylalgia is a pain condition of the upper extremity which can affect 1–3% of the general population and has a peak incidence ranging between the ages of 35 and 54 years [1]. Lateral epicondylalgia is traditionally considered an overload tendinopathy of the wrist extensor musculature associated with changes in the pain system and impairments in the motor system [2]. Although the etiology of lateral epicondylalgia is not properly understood, there is clear evidence of the presence of altered nociceptive processing [3]. The presence of altered nociceptive processing is supported by the presence of bilateral pressure hyperalgesia at the affected area (elbow), at a segmental-related area (cervical spine) and at remote pain-free areas (small evidence) [4]. However, most studies have investigated pressure pain hyperalgesia over tendon or muscle tissues [4].

One often-forgotten anatomical structure that may be involved in lateral epicondylalgia is the radial nerve as this nerve innervates the lateral part of the elbow and the wrist extensor muscles and runs intimately close to the extensor carpi radialis brevis muscle when passing throughout the arcade of Frohse [5]. Bordachar has proposed that elbow-related pain may originate in and propagate from any of the structures related to the radial nerve or even from the nerve itself [6]. This assumption is based on studies suggesting the presence of nerve hypersensitivity in people with elbow pain. For instance, two old studies observed a positive response to a neurodynamic test of the radial nerve in patients suffering from lateral epicondylalgia [7,8]. Fernández-de-las-Peñas et al. found bilateral hyperalgesia to pressure pain (as expressed by lower pressure pain thresholds) over the radial nerve in a small sample (*n* = 17) of women with unilateral lateral epicondylalgia [9].

More recent studies, using ultrasound imaging, have also identified changes at the radial nerve. Gürçay et al. observed a larger cross-sectional area of the radial nerve (swelling in the nerve sheath), without electromyographic changes, on the symptomatic side in patients with unilateral refractory lateral epicondilalgia [10]. Abhimanyu et al. also found that the increased thickness in the radial nerve within the symptomatic side (just found in a proportion of patients) was associated with higher related disability in people with lateral epicondylalgia [11]. De la Cruz Torres also found higher cross-sectional area of the radial nerve on the affected side but also decreased excitability of both radial nerves bilaterally in patients with unilateral chronic lateral epicondylalgia [12]. These previous studies did not include a control group using the asymptomatic side as comparison.

Since people with lateral epicondylalgia can exhibit bilateral changes as a manifestation of hyper-excitability of the central nervous system, studies including a control pain-free group are needed. Additionally, no previous study has investigated if the presence of morphological changes within the radial nerve is associated with pressure pain nerve hyperalgesia. Accordingly, the aims of the current study were: (1) to investigate the presence of pressure pain hyperalgesia over the radial nerve in people with unilateral lateral epicondylalgia; (2) to identify the presence of nerve swelling (increased cross-sectional area), as assessed using ultrasound imaging, on the radial nerve in people with lateral epicondylalgia; and (3) to investigate if an association exists between pressure pain sensitivity and the cross-sectional area of the radial nerve in individuals with lateral epicondylalgia. We hypothesized that patients with unilateral epicondylalgia would exhibit bilateral hyperalgesia to pressure pain and higher cross-sectional area at the radial nerve as compared to healthy pain-free subjects. In addition, it was also expected that a linear association between cross-sectional area and pressure pain thresholds would be observed in the lateral epicondylalgia group.

## 2. Methods

### 2.1. Study Design

A cross-sectional case–control study following the Strengthening the Reporting of Observational studies in Epidemiology (STROBE) guidelines was conducted [13]. The Local Ethics Committee of Universidad Rey Juan Carlos (nº 1801202102321) approved the study. All participants signed written informed consent prior to their inclusion.

### 2.2. Participants

Consecutive subjects suffering from lateral elbow pain presenting to a physical therapy clinic in Madrid (Spain) were screened for eligible criteria. Subjects underwent a physical examination, conducted by an experienced physical therapist to assess inclusion/exclusion criteria. Patients were included if at least three of the following criteria were identified during physical examination: (1) pain over the lateral aspect of the elbow; (2) pain on palpation over the lateral epicondyle and/or the common wrist extensors tendon; (3) elbow pain appearing or increasing with hand gripping; or (4) elbow pain appearing or increasing with resisted static contraction or stretching of the wrist extensors. Symptoms had to be present for at least 3 months and had to be unilateral only.

In addition, age- and sex-matched healthy pain-free subjects were also recruited from local announcements. To be included in this group, subjects could not have reported a previous history of lateral elbow pain and no pain symptoms in the upper extremity over the previous year.

The exclusion criteria for both groups were: (1) bilateral elbow pain; (2) older than 65 years of age; (3) previous steroid injections on the elbow; (4) previous surgery in the upper extremity; (5) multiple diagnoses in the upper extremity (cervical radiculopathy); (6) history of upper extremity or neck trauma (whiplash); or (7) comorbid medical condition (e.g., rheumatoid arthritis or fibromyalgia).

### 2.3. Pain and Function Outcomes

An 11-point Numeric Pain Rating Scale (NPRS; 0: no pain, 10: maximum pain) was used to assess pain the mean intensity of elbow pain experienced in the preceding week [14]. Further, the Patient-Rated Tennis Elbow Evaluation (PRTEE) was used to assess elbow-related function [15,16]. The questionnaire consists of 2 parts including both pain and function. The first part includes 5 questions scored from 0 (no pain) to 10 (most severe pain). The scores for these questions are summed, providing a score over 50 points. The second part includes 10 questions about function. The scores for these questions are summed and divided by 2 providing a total score over 50 points. Both subscales are summed, and a total PRTEE score out of 100 is reported. Lower scores indicate better function [15,16].

### 2.4. Pressure Pain Thresholds

**Figure 1:**
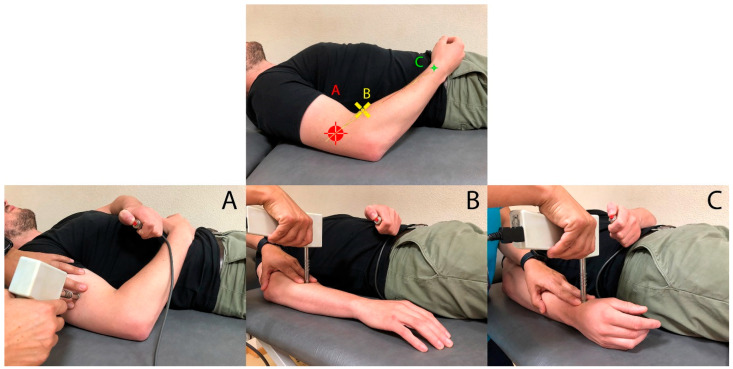
Pressure pain threshold (PPT) points of assessment on the radial nerve. (**A**) Spiral groove; (**B**) arcade of Frohse; (**C**) anatomic snuffbox.

Pressure pain thresholds (PPTs), the amount of pressure needed to change the sensation of pressure to pain, were assessed with an electronic algometer (Somedic^®^ Algometer, Sollentuna, Sweden) over different points along the anatomical path of the radial nerve. Pressure was applied at a rate of approximately 30 kPa/s on each point. Participants were trained to press the “stop” button as soon as they first felt the sensation change from pressure to pain. Three trials were applied on each point, with a resting period of 30 s between each trial to avoid temporal summation [17], and the mean was calculated and used in the main analysis. Pressure pain thresholds were assessed over the radial nerve at the following points as the reliability has been found to be excellent (ICC 0.98–0.99) in patients and good to excellent (ICC 0.74–0.99) in healthy subjects [18]:

Spiral groove (Figure 1A): the radial nerve was identified where it passes through the lateral intermuscular septum between the medial and lateral heads of triceps to enter the mid- to lower third of the humerus (spiral groove) [19].

Arcade of Frohse (Figure 1B): The arcade of Frohse is located in the radial region, 5 cm distal to the lateral epicondyle above the radial head.

Anatomic snuffbox (Figure 1C): This region is sensory-innervated by the superficial branch of the radial nerve. The algometer was perpendicularly placed at a point located at trapezio-metacarpal joint at the bottom of the anatomic snuffbox.

### 2.5. Ultrasound Imaging Acquisition Protocol

**Figure 2:**
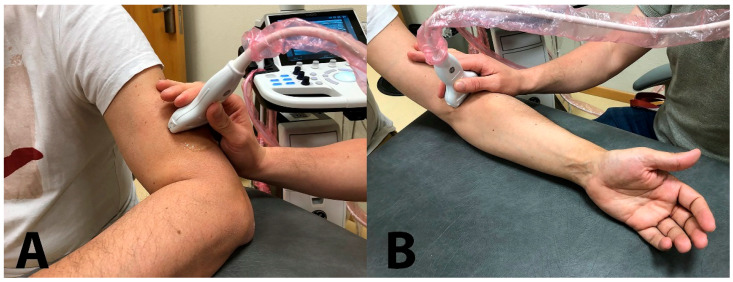
Ultrasound probe location for evaluation of the cross-sectional area of the radial nerve at the spiral groove (**A**) and the antecubital fossa (**B**).

**Figure 3:**
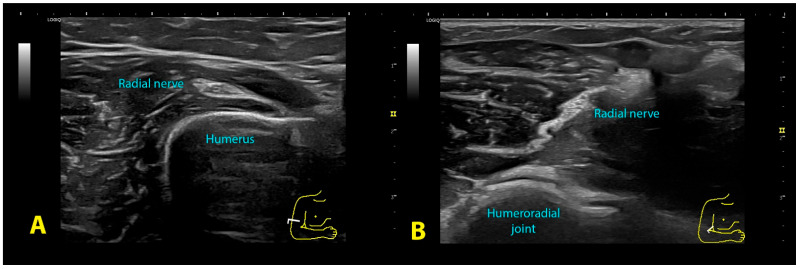
Ultrasound image of the radial nerve at the spiral groove (**A**) and the antecubital fossa (**B**).

All ultrasound images were acquired with a GE Logiq P9 device and a linear 6–15 MHz transducer ML-6–15-D (General Electric Healthcare, Milwaukee, WI, USA). The console settings were also standard for all the acquisitions (Frequency = 12 MHz, Gain = 65 dB and Depth = 3 cm). All measurements were conducted following the European Society of Musculoskeletal Radiology guidelines [20]. The radial nerve was evaluated at two different points along its anatomical path as in previous studies [10,11]:

Spiral groove: The patient was sitting with their arm resting on table, elbow flexed 90° with the forearm pronated and hand relaxed resting on table. The probe was placed transverse to the axis of the arm, on the lateral aspect of the distal third of the humerus (Figure 2A), searching for the intermuscular septum and the exit of the nerve from the radial groove (Figure 3A).

Antecubital fossa: The patient was sitting with the arm resting on the table, the elbow extended and the forearm supinated. The probe was placed transverse to the axis of the arm at the level of the anterior aspect of the elbow flexure, observing the elbow joint space (Figure 2B). The nerve lies between the brachioradialis muscle and the anterior brachialis muscle (Figure 3B). The radial nerve was imaged before it divided into its two branches; therefore, sometimes it was necessary to move the probe 1–2 cm above the elbow joint line.

### 2.6. Ultrasound Measurement

An independent researcher codified, saved, and, after exporting all the images acquired to a DICOM format, sent the files to the examiner. All images were analyzed using the ImageJ offline DICOM software 1.8^®^ (National Institute of Health, Bethesda, MD, USA, v.1.53a). For each point, three measurements were made, then the mean was obtained and used in the comparative analysis. The cross-sectional area of the radial nerve was calculated as follows [21]:

Intraneural cross-sectional area was calculated by tracing a continuous line around the inner borders of the hyperechoic rim, excluding connective tissue, the epineurium, that surrounds the nerve.

Nerve cross-sectional area was calculated by tracing a continuous line around the surrounding connective tissue (around the outer edge of the hyperechoic line).

**Figure 4:**
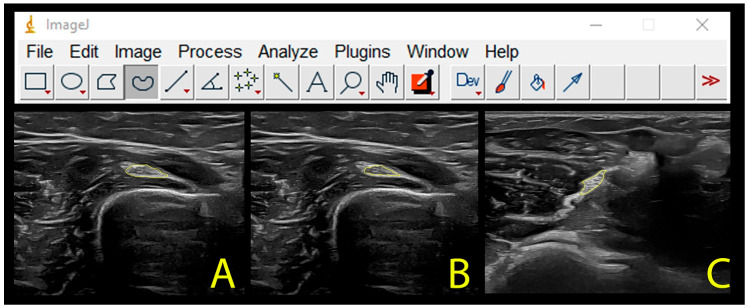
Calculation of the cross-sectional area of the radial nerve for the total nerve (**A**) or intraneural (**B**) at the spiral groove and for the antecubital fossa (**C**).

Nerve (Figure 4A) and intraneural (Figure 4B) cross-sectional areas were assessed at the spiral groove, whereas nerve cross-sectional area was just assessed at the antecubital fossa (Figure 4C).

A study on the reliability of ultrasound measures was conducted with images from 10 subjects not included in the main analysis. Intra-examiner reliability was calculated from examiner one evaluating the same images twice, one month apart. Inter-examiner reliability was calculated with two examiners evaluating the same images once each. Intra-class correlation coefficients (ICC3,1 for intra-examiner reliability and ICC3,2 for inter-examiner reliability, calculated with a 2-way mixed model, consistency type) were calculated. Intra-rater reliability was excellent (ICC3,1: 0.988, 95% CI 0.969–0.995) whereas inter-rater reliability was good (ICC3,2: 0.823, 95% CI 0.552–0.930).

### 2.7. Sample Size Calculation

The sample size calculation was powered for both outcomes (e.g., PPTs or cross-sectional area) separately to determine the best approximation. For PPTs, the sample size determination was based on detecting a moderate–large effect size of 0.75 between patients and controls, a 2-tailed test, with an alpha level (α) of 0.05, and a desired power (β) of 90%. This determination generated a sample size of at least 30 participants per group.

For the cross-sectional area assessment, the sample size determination was based on detecting an expected between-group difference of 1mm thickness, with a standard deviation of 1.5 mm, power (β) of 90%, and with an alpha level (α) of 0.05. This determination generated a sample size of at least 35 participants per group.

### 2.8. Statistical Analysis

Statistical analysis was performed using SPSS software version 20.0 (Chicago, IL, USA). A normal distribution of the data was verified using the Shapiro–Wilk test. A two-way analysis of covariance (ANCOVA) with side (affected/unaffected or dominant/non-dominant) as the within-group factor, group (patients or controls) as the between-subject factor, and gender as the covariate factor was used to determine differences in PPTs and cross-sectional area on each point (spiral groove or antecubital fossa). Post hoc comparisons were conducted with the Bonferroni test. Finally, Pearson correlation tests (r) were used to determine the association between pain, function, PPTs and cross-sectional area of the radial nerve. The statistical analysis was conducted at a 95% confidence level, and a P-value less than 0.05 was considered statistically significant.

## 3. Results

### 3.1. Participants

From a sample of 45 subjects with lateral elbow pain screened for eligible criteria, a total of 37 patients (43% women, age: 45.5 ± 9.5 years; height: 1.71 ± 0.1 m; weight: 72 ± 12.5 kg; BMI: 24.5 ± 2.8 kg/cm^2^) were included. Eight (18%) subjects were excluded for the following reasons: bilateral symptoms (*n* = 4), previous whiplash (*n* = 2), previous corticoid injection (*n* = 1), and diagnosis of cervical radiculopathy (*n* = 1). Patients exhibited a mean history of 13.5 (SD 5) months with pain symptoms, a mean pain intensity at rest of 4.8/10 (SD 1.2), and a PRTEE score of 52.4/100 (SD 17.4) points.

The control group consisted of 37 age- and sex-matched pain-free controls (43% women, age: 45.0 ± 9.0 years; height: 1.72 ± 0.1 m; weight: 72 ± 10.5 kg; BMI: 24.4 ± 2.7 kg/cm^2^.

### 3.2. Pressure Pain Thresholds of the Radial Nerve

The ANCOVA revealed significant differences between both groups (spiral groove: F = 7.144, *p* < 0.001; arcade of Frohse: F = 10.816, *p* < 0.001; anatomic snuffbox; F = 37.525, *p* < 0.001) and sides (spiral groove: F = 9.030, *p* = 0.003; arcade of Frohse: F = 5.385, *p* = 0.022; anatomic snuffbox; F = 4.087, *p* = 0.045) for PPTs at all points. Additionally, significant group * side interactions (spiral groove: F = 5.968, *p* = 0.016; arcade of Frohse: F = 4.381, *p* = 0.038; anatomic snuffbox; F = 8.349, *p* = 0.004) were also observed: individuals with lateral epicondylalgia exhibited lower PPTs on the radial nerve of the affected side as compared with the unaffected side (*p* < 0.01) and both sides in healthy controls (*p* < 0.001). Table 1 details the PPTs assessed over the radial nerve at each point within each group.

In addition, the ANCOVA showed a significant interaction of gender for PPT over all points: spiral groove: F = 8.622, *p* = 0.004; arcade of Frohse: F = 5.929, *p* = 0.016; anatomic snuffbox; F = 7.784, *p* = 0.006): females exhibited lower PPTs than males in both groups for the three nerve points.

### 3.3. Cross-Sectional Area of the Radial Nerve

The ANCOVA revealed significant differences between groups for the cross-sectional area of the nerve at both sites (spiral groove: F = 6.067, *p* = 0.015; antecubital fossa: F = 17.449, *p* < 0.001), but not for the intraneural cross-sectional area at the spiral groove (F = 0.003, *p* = 0.959). Additionally, a significant effect of side (spiral groove nerve: F = 13.806, *p* < 0.001; spiral groove intraneural: F = 8.886, *p* = 0.003; antecubital fossa: F = 18.273, *p* < 0.001) and group * side interaction (spiral groove nerve: F = 21.752, *p* < 0.001; spiral groove intraneural: F = 4.395, *p* = 0.038; antecubital fossa: F = 21.906, *p* < 0.001) in all assessed points was observed: individuals with lateral epicondylalgia exhibited higher cross-sectional area on the radial nerve (total and intraneural) of the affected side as compared with the unaffected side (*p* < 0.01) and both sides in healthy controls (*p* < 0.001).

No significant effect of gender for the cross-sectional area of the radial nerve was observed (spiral groove nerve: F = 0.417, P = 0.520; spiral groove intraneural: F = 0.188, *p* = 0.665; antecubital fossa: F = 0.684, P = 0.410). Table 2 summarizes cross-sectional areas over the radial nerve at each point within each group.

### 3.4. Associations

**Figure 5:**
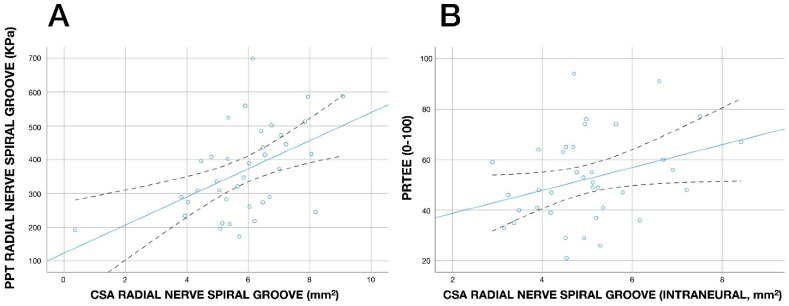
Scatterplot of the correlations between pressure pain thresholds (PPTs, kPa) over the radial nerve at the spiral grove and cross-sectional area (CSA, mm^2^) of the radial nerve at the spiral groove (**A**) and between the Patient-Rated Tennis Elbow Evaluation (PRTEE. 0–100) and cross-sectional area (CSA, mm) of the radial nerve (intraneural) at the spiral groove (**B**) in individuals with unilateral lateral epicondylalgia (*n* = 37). Note that some points can be overlapping. A positive linear regression line is fitted to the data (continuous line). The dashed lines represent the confidence intervals.

A significant negative association between the cross-sectional area of the radial nerve at the spiral groove with PPT on the radial nerve at the spiral groove (r = −0.496, *p* = 0.002, Figure 5A) was observed: the greater the cross-sectional area (nerve swelling), the lower the PPT (the higher the pressure pain sensitivity). No other significant association between PPTs and cross-sectional areas was observed. In addition, a significant positive association between cross-sectional area of the radial nerve (intraneural) at the spiral groove with PRTEE score (r = 0.325, *p* = 0.045, Figure 5B) was also identified: the greater the cross-sectional area (nerve swelling), the higher the PRTEE score (higher disability).

## 4. Discussion

The current study revealed generalized pressure pain hyperalgesia (lower PPTs) and also nerve swelling (increased cross-sectional area) of the radial nerve on the symptomatic/affected side in individuals with unilateral lateral epicondylalgia. These results could express the presence of hyperalgesia of the nerve tissue, suggesting that the radial nerve could represent a peripheral drive to initiate and maintain altered pain processing in lateral epicondylalgia.

In this study, PPTs were found to be significantly decreased over the radial nerve on the symptomatic side at three different points along its anatomical path in individuals with unilateral lateral epicondylalgia. Pressure pain hyperalgesia of the radial nerve on the symptomatic side was expressed as side-to-side differences of 100 kPa, representing a meaningful difference. We also identified nerve swelling on the affected side as expressed as increased cross-sectional area of the radial nerve. Our results are similar to those previously observed by Gürçay et al. [10] and Abhimanyu et al. [11] who also observed nerve swelling in the radial nerve of individuals with lateral elbow pain; however, neither study included a control pain-free group. Previous and current results support the presence of nerve swelling in this population. Won et al. reported cross-sectional area for the radial nerve at the spiral groove of 4.6 (SD 0.9) in healthy subjects [21], data similar to our control group for intraneural tissue. Accordingly, it could be assumed that between-groups differences were real, since it reached 1–2 mm of difference between the radial nerve of the affected side when compared with the non-symptomatic side or healthy controls. In fact, the presence of radial nerve swelling could explain why some individuals with lateral epicondylalgia exhibit similar symptomatology as those with radial tunnel syndrome, a dynamic/intermittent compression neuropathy of the radial nerve [22], and that some patients with lateral epicondylalgia also suffered from radial tunnel syndrome [23]. Nevertheless, it should be recognized that all the assessed points were located based on anatomical landmarks related to the radial nerve and between-individuals differences could exist.

Bordachar proposed a model for lateral epicondylalgia where the neural tissue, e.g., the radial nerve can be involved [6]. This model suggested the following three steps in the potential development of lateral epicondylalgia [6]: (1) a stimulus increasing nociceptors neuronal activity (e.g., repetitive microtrauma in the elbow); (2) an innervated tissue, susceptible of being sensitized (e.g., wrist extensor muscles/tendon), and (3) a connecting nerve between the target peripheral tissues and the central nervous system (e.g., the radial nerve). This model is based on the premise that the altered nociceptive pain processing observed in patients with chronic pain is mostly associated with long-lasting nociceptive afferences from peripheral tissue. Most theories mainly propose muscle or tendon tissues for explaining lateral epicondylalgia-related pain [2]; however, these theories do not consider the nerve tissue. Our findings would suggest that nociception from nerve tissues, i.e., the radial nerve, and not just that from the wrist extensor muscles/tendon, can also be involved in lateral epicondylalgia-related pain since nerve tissue may become irritated by inflammatory processes and may sensitize C-fiber nociceptors of the “nervi nervorum” (nerves that innervate the connective tissue layers of the nerve itself). In a sensitized state, nerve endings of the “nervi nervorum” can lead to an increase in the synthesis and release of algogenic substances, resulting in neurogenic inflammation and spontaneous discharges in the nerve fibers. Therefore, the radial nerve could represent a peripheral drive to initiate and maintain altered pain processing in this population [24]. Nevertheless, this hypothesis does not assume that lateral epicondylalgia is a neuropathic condition; it proposes that nerve tissue could also contribute to the altered nociceptive processing observed in lateral epicondylalgia.

It is also possible that the generalized hyperalgesia observed over neural tissue could be evoked by the central mechanisms as a result of an increased responsiveness of nociceptive neurons to non-noxious stimuli [25]. In fact, the generalized sensitisation of neural tissues is considered a sign of a hyper-excitability state of the central nervous system and it has been found in pain conditions of musculoskeletal origin such as plantar heel pain [26] or tension-type headache [27]. However, the fact that we observed hypersensitivity to pressure pain just on the symptomatic side would suggest that a more peripheral mechanism is involved in creating nerve hypersensitivity to pressure pain in people with lateral epicondylalgia. Additionally, we only evaluated the pressure sensitivity of the radial nerve, the nerve innervating the wrist extensor musculature, so our results do not support the presence of widespread pressure pain sensitivity of other nerve tissues, e.g., median or ulnar nerve, in lateral epicondylalgia.

The results from this study have several clinical implications. First, if lateral elbow pain can be reproduced through a mechanical provocation of the neural structures, as can be performed with neurodynamic tests, it may also be possible to relieve symptomatology through the treatment of such structures. In such a scenario, interventions targeting the nerve tissue can perhaps be applied for the management of lateral epicondylalgia pain. In fact, evidence suggests that radial nerve surgical release can be effective in patients with recalcitrant lateral elbow pain [28]. Further, it is also plausible that nerve-biased interventions can be applied as a complement to current treatment strategies used for managing lateral epicondylalgia. For instance, a meta-analysis found low-to-moderate evidence for a positive effect of the application of dry needling for pain and related disability in the short-term in people with lateral epicondylalgia of musculoskeletal origin [29]. It is possible that the radial nerve, and not just the muscle/tendon tissue, needs to be treated in some patients, perhaps explaining the lack of effectiveness of some current interventions. In those individuals with lateral epicondylalgia exhibiting a neural component, treatments targeting the nerve tissues, i.e., percutaneous nerve stimulation [30], could be applied for pain and related disability by decreasing nerve sensitivity [31] and nerve swelling. Supporting this hypothesis, a pilot clinical trial recently observed that the application of percutaneous nerve stimulation targeting the radial nerve was effective for pain and related disability in a small sample of patients with lateral epicondylalgia [32]. Future clinical trials are needed to confirm this hypothesis.

Finally, we should recognize some potential limitations to this case–control study. First, the cross-sectional design did not permit us to determine a cause-and-effect relationship between the observed findings and the evolution of LE. Second, although we calculated the sample size, studies with larger sample sizes are needed to further confirm current results. Third, we did not include psychological outcomes, e.g., mood disorders or kinesiophobia, which could exert an effect on pressure pain sensitivity.

## 5. Conclusions

The results of the current study demonstrated hyperalgesia to pressure pain, as expressed by lower PPTs, and nerve swelling, as expressed by increased cross-sectional area, of the radial nerve at the symptomatic side in people with lateral epicondylalgia. No direct association between PPTs and nerve swelling was found. These results could express the presence of hyperalgesia of the nerve tissue, suggesting that the radial nerve can represent a peripheral drive to initiate and maintain altered pain processing in individuals with lateral epicondylalgia.

## Figures and Tables

**Table 1:** Differences in pressure pain thresholds (PPTs) over the radial nerve between patients with lateral epicondylalgia and healthy controls.

	Spiral Groove ^#^	Arcade of Frohse ^#^	Anatomical Snuffbox ^#^
Patients with Lateral Epicondylalgia			
Affected side	366.9 (130.5) kPa	378.9 (151.9) kPa	435.5 (123.2) kPa
	(95% CI 323.4–410.5)	(95% CI 328.3–429.6)	(95% CI 394.4–476.5)
Non-affected side	489.7 (156.6) kPa	487.2 (180.9) kPa	547.0 (159.3) kPa
	(95% CI 437.5–541.9)	(95% CI 426.9–547.6)	(95% CI 493.9–600.1)
Healthy Controls			
Dominant side	482.2 (132.5) kPa	510.9 (121.9) kPa	640.2 (139.4) kPa
	(95% CI 438.1–526.4)	(95% CI 470.3–551.6)	(95% CI 593.8–686.7)
Non-dominant side	494.9 (126.7) kPa	516.6 (135.5) kPa	620.5 (128.0) kPa
	(95% CI 452.7–537.2)	(95% CI 471.4–561.8)	(95% CI 577.8–663.2)

Values (kPa) are expressed as mean (standard deviation) (95% confidence interval). ^#^ Significant differences between patients and controls (two-way ANCOVA test).

**Table 2:** Differences in cross-sectional area (CSA) of the radial nerve in the spiral groove (nerve and intraneural) and antecubital fossa (nerve) between patients with lateral epicondylalgia and healthy controls.

	Spiral Groove		Antecubital Fossa
	Radial Nerve ^#^	Intraneural ^#^	Radial Nerve ^#^
Patients with lateral epicondylalgia			
Affected side	10.85 (1.88) mm^2^	5.01 (1.25) mm^2^	12.35 (1.90) mm^2^
	(95% CI 10.25–11.50)	(95% CI 4.60–5.45)	(95% CI 11.72–13.00)
Non-affected side	8.65 (1.42) mm^2^	4.13 (0.95) mm^2^	9.52 (1.31) mm^2^
	(95% CI 8.20–9.15)	(95% CI 3.80–4.45)	(95% CI 9.08–9.95)
Healthy Controls			
Dominant side	9.00 (1.75) mm^2^	4.65 (1.25) mm^2^	9.80 (1.52) mm^2^
	(95% CI 8.40–9.60)	(95% CI 4.22–5.01)	(95% CI 9.30–10.30)
Non-dominant side	9.25 (1.25) mm^2^	4.49 (0.85) mm^2^	9.90 (1.50) mm^2^
	(95% CI 8.82–9.65)	(95% CI 4.20–4.75)	(95% CI 9.40–10.40)

Values (mm^2^) are expressed as mean (standard deviation) (95% confidence interval). ^#^ Significant differences between patients and controls (two-way ANCOVA test).

## Data Availability

All data derived from this study are presented in the text.
